# College and Hospital Notes

**Published:** 1906-08

**Authors:** 


### COLLEGE AND HOSPITAL NOTES.

THE NEW YORK POST-GRADUATE MEDICAL SCHOOL AND HOSPITAL has issued its Twenty-fifth Annual Announcement which is for the school year of 1906-7 that begins October 1, 1906. The directors invite the attention of the medical profession of this country, Canada, and the West Indies to the advantages possessed by this school for post-graduate instruction, in which is taught every branch of medicine and surgery with a scientific purpose to instruct those who attend, so they may be able to do themselves whatever they see the instructors do. The equipment is complete as to material and ability of the faculty and nowhere in the world is more conscientious or more valuable instruction given. Every physician who would keep pace with medical progress should spend a few weeks in each year at this school.

---

THE KING EDWARD VII SANATORIUM FOR CONSUMPTIVES has been built at Midhurst, and lately was opened by their Majesties, King Edward and Queen Alexandra. The money cost of the institution was one million dollars and its equipment is probably the finest of its kind in the world. Its location in Sussex on the South Downs, is superb; it is six hundred feet above sea level, and is twelve miles inland; the grounds include about one hundred and fifty acres, the buildings and terraced gardens being sheltered on the North by pine woods. Accomodations have been made for one hundred patients, each provided with 1,730 cubic feet of air in their sleeping rooms. The walls resemble porcelain and their sanitary finish is perfect. Without doubt this splendid charity is unequalled, and it reflects the highest credit to Great Britain.

